# Microstructure and Mechanical Properties of W-Al_2_O_3_ Alloy Plates Prepared by a Wet Chemical Method and Rolling Process

**DOI:** 10.3390/ma15227910

**Published:** 2022-11-09

**Authors:** Changji Wang, Xiaonan Dong, Yao Liu, Shizhong Wei, Kunming Pan, Cheng Zhang, Mei Xiong, Feng Mao, Tao Jiang, Hua Yu, Xiaodong Wang, Chong Chen

**Affiliations:** 1 School of Materials Science and Engineering, Henan University of Science and Technology, Luoyang 471000, China; 2 Henan Key Laboratory of High-Temperature Structural and Functional Materials, Henan University of Science and Technology, Luoyang 471003, China; 3 Longmen Laboratory, Luoyang 471000, China; 4 Scientific Research Platform Service Center of Henan Province, Zhengzhou 450003, China

**Keywords:** ODS-W, wet chemical method, Al_2_O_3_-reinforced, tungsten alloy plates

## Abstract

The uneven distribution and large size of the second phase weakens the effect of dispersion strengthening in ODS-W alloys. In this article, the W-Al_2_O_3_ composite powders were fabricated using a wet chemical method, resulting in a finer powder and uniformly dispersed Al_2_O_3_ particles in the tungsten-based alloy. The particle size of the pure tungsten powder is 1.05 μm and the particle size of W-0.2 wt.%Al_2_O_3_ is 727 nm. Subsequently, the W-Al_2_O_3_ alloy plates were successfully obtained by induction sintering and rolling processes. Al_2_O_3_ effectively refined grain size from powder-making to sintering. The micro-hardness of the tungsten alloy plates reached 512 HV_0.2_, which is 43.7% higher than that of pure tungsten plates. The nano-hardness reached 14.2 GPa, which is 24.1% higher than that of the pure tungsten plate; the compressive strength reached 2224 MPa, which is 37.2% higher than that of the pure tungsten.

## 1. Introduction

Tungsten or tungsten-base alloys are used in the aerospace industry to make nozzles for rocket propulsion and armor-piercing warheads in the defense military industry owing to a high melting point (3420 °C), high density, excellent thermal shock resistance, and high-temperature strength [1]. Because of the excellent sputtering resistance and low tritium retention, tungsten is also considered to be the most ideal material for use in the first wall to resist fusion plasma irradiation in the nuclear industry [2,3,4,5,6]. However, the room temperature brittleness, irradiation embrittlement, and recrystallization embrittlement of tungsten limit its wider applications [7].

The introduction of oxides and carbides into the tungsten matrix is considered to be an effective method to solve the above problems of tungsten [8,9,10,11]. Oxides such as La_2_O_3_ [12], Y_2_O_3_ [13], ZrO_2_ [14], and Al_2_O_3_ [15] have been widely studied. Al_2_O_3_ has been added to metal matrix composites (such as Al, Mo, and Mg) as a strengthening phase because of the excellent high-temperature properties, good chemical stability, and low cost [16,17,18,19]. The introduction of a second phase using conventional mechanical alloying methods not only results in a non-uniform distribution of the second phase but also tends to introduce impurities during the alloying process; the use of wet chemical methods was able to circumvent these problems and prepare powders to the nanoscale [20,21,22]. In our previous work, the addition of finer Al_2_O_3_ to tungsten alloys using a wet chemical method was found to significantly improve hardness and toughness [15] and increase the recrystallization temperature of the alloys [23].

This study used a wet chemical method to achieve a homogeneous distribution of the second phase Al_2_O_3_ in the alloy. The alloy powder prepared using this method also allows for effective grain refinement, resulting in significant improvements in the properties and machinability of the alloy. The tungsten alloys were unidirectionally rolled into plates, and the mechanical properties of the tungsten alloy plates were investigated by conducting nano-hardness, micro-hardness, and room-temperature compression tests. The effects of the microstructure and weaving on the mechanical properties of the alloy plates were studied by performing scanning electron microscopy (SEM), electron backscatter diffraction (EBSD), and transmission electron microscopy (TEM).

## 2. Materials and Methods

The W-Al_2_O_3_ composite powders were prepared from aluminum nitrate (Al(NO_3_)_3_) and ammonium para tungstate ((NH₄)10W_12_O_41_·5H₂O). Aluminum nitrate (Al(NO_3_)_3_) was dissolved in deionized water by stirring using a magnetic mixer. The obtained solution was added to a concentrated nitric acid solution to adjust the pH (pH = 0.5). The treated solution was poured into a hydrothermal reactor and placed in a drying oven for hydrothermal heating (170 °C, 15 h). Ammonium para tungstate was added to deionized water and dissolved completely. After the hydrothermal reaction, the precursor solution of Al_2_O_3_ was poured into the aqueous solution of ammonium para tungstate by applying magnetic stirring + ultrasonic shaking (20–24 h). The evenly stirred mixture was poured into a vessel and placed in a drying oven (80 °C). The dried composite powder was crushed using a pulverizer. The crushed powder was placed in a muffle furnace and calcined (500 °C, 2 h) to obtain the composite powder of WO_3_ and Al_2_O_3_. The composite powder was placed in a hydrogen reduction furnace for two-stage reduction (620 °C, 6 h, 920 °C, 8 h). Finally, the W-Al_2_O_3_ composite powders were obtained. The W-Al_2_O_3_ composite powders were loaded into a rubber mold and rolled into a square billet by employing cold isostatic pressing (CIP). The billets were placed in an induction sintering furnace at a temperature of 2350 °C for a sintering time of 8 h. The hydrogen atmosphere was maintained throughout the sintering process to prevent oxidation of the alloy. The sintered body with a thickness of 24 mm was heated to 1500–1600 °C in the heating furnace and the tungsten alloy plates with a thickness of 3 mm were finally obtained after several unidirectional rolling. The total thickness reduction after rolling was 87.5%. Five compositions of tungsten alloy plates with Al_2_O_3_ contents of 0, 0.2 wt.%, 0.4 wt.%, 0.6 wt.%, and 0.8 wt.% were prepared to investigate the effect of different contents of Al_2_O_3_ on the properties and microstructure of the plates.

**Figure 1:**
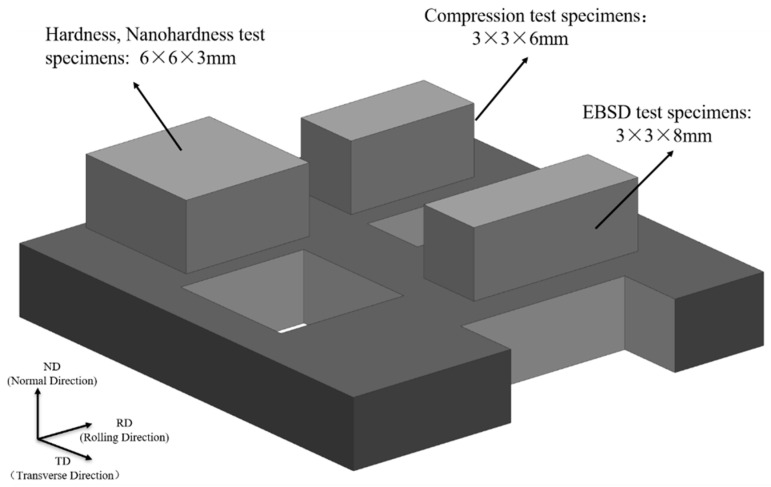
Schematic diagram of test specimens cut from the tungsten alloy plates.

The physical phase of the prepared alloy powder was examined by performing X-ray diffraction (Bruker-D8) equipped with a CuKα target. The voltage and current used in XRD experiments were 40 kV and 40 mA, respectively. The microscopic morphology and the distribution of Al_2_O_3_ in the W-Al_2_O_3_ composite powder were analyzed by using JEOL JSM-IT 800 SHL scanning electron microscope, and the particle size of the W-Al_2_O_3_ composite powder was tested by using an LS-909 laser particle sizer (Zhuhai OMEC Instruments Co., Ltd., Zhuhai, China). After rolling the alloy, samples (6 mm × 6 mm) were cut from the rolled surface. After mounting and polishing, the micro-hardness of the alloy plates was tested using an HV-1000 Vickers micro-hardness tester (Laizhou Huayin Test Instrument Co. Ltd., Laizhou, China) with a load of 200 g and a holding time of 15 s. Each sample was tested 20 times, then the maximum and minimum values were removed, and the remaining values were averaged, which was conducted to minimize error. The nano-indentation hardness was characterized using a G200 Nano-Indenter instrument (Keysight Technologies) equipped with a Berkovich indenter. In order to ensure the accuracy of the test data, the tungsten samples were polished using the diamond suspensions and 36 points were tested for each sample under the constant strain rate of 0.05 s^−1^. The pressing depth and dwell time were 1500 nm and 15 s, respectively. The maximum load in the nanoindentation tests was 870 mN. The hardness of tungsten alloys was measured by continuous stiffness method (CSM) and calculated by Oliver–Pharr method. Room-temperature compression samples (3 mm × 3 mm × 6 mm) were cut along the rolling direction of the plates, and room-temperature compression experiments were performed in a Instron 5582 Double Column Electronic Universal Testing Machine at a rate of 0.5 mm/min. Samples (3 mm × 8 mm) were cut in the tangential direction of the plates and polished in an argon-ion polishing machine (IB-19530CP, JEOL, Akishima-shi, Tokyo, Japan). After the sample was set, smoothed, and polished by using an argon-ion polisher, the weaving and microstructure of the deformed plates were observed using a JEOL JSM-IT 800 SHL scanning electron microscope (Akishima-shi, Tokyo, Japan) and an Oxford c-nano EBSD system with an EBSD scan step of 160 nm. Analysis of the interface and microstructure of the rolled sheet using FEI Talos F200X Transmission electron microscope. Figure 1 shows the schematic diagram of the examined sample.

## 3. Results and Discussion

### 3.1. Powder Characterization

**Figure 2:**
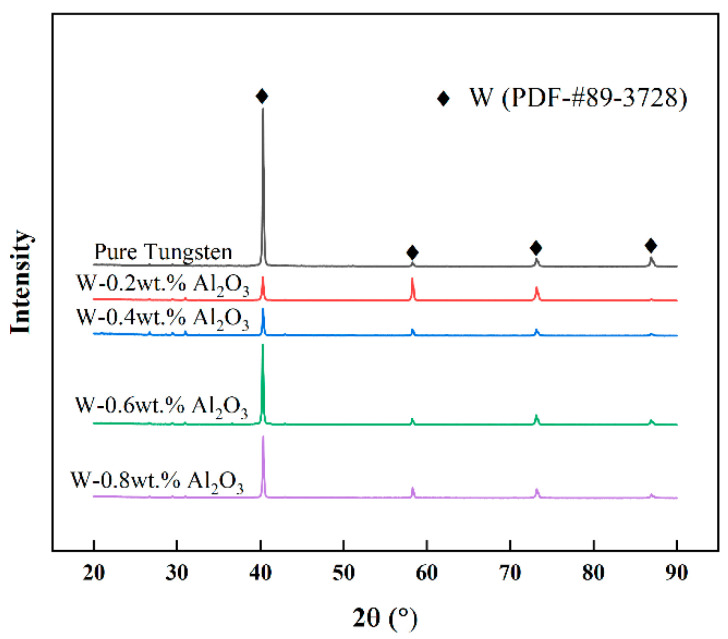
XRD patterns of the composite powders.

Figure 2 shows the XRD patterns of the composite powders. The XRD peaks of pure tungsten and W-Al_2_O_3_ alloy powders with different compositions are the same, with no diffraction peaks of other phases except those of tungsten; this may be because the content of Al_2_O_3_ is extremely low to be detected in XRD. In addition, the intensity of the diffraction peaks is slightly different; this may be because the crystallinity of tungsten is affected by the addition of Al_2_O_3_.

**Figure 3:**
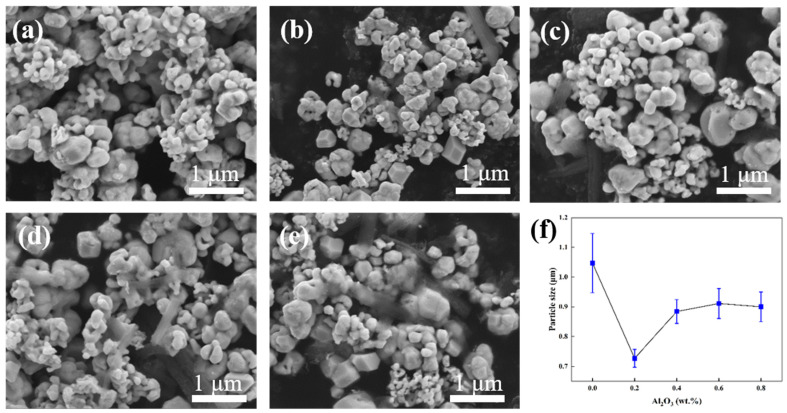
SEM images of the composite powders: (**a**) pure tungsten, (**b**) W-0.2 wt.%Al_2_O_3_, (**c**) W-0.4 wt.%Al_2_O_3_, (**d**) W-0.6 wt.%Al_2_O_3_, (**e**) W-0.8 wt.%Al_2_O_3_, (**f**) grain size of the composite powders.

Figure 3 shows the SEM images of the composite powders with different compositions. The average particle size of the pure tungsten powder is 1.05 μm, as shown in Figure 3a. The particles appear to be bonded and the particles are significantly larger than those of the composite powders with other compositions. When the content of Al_2_O_3_ is 0.2 wt.% (Figure 3b), the particles are better than those of the powders with other compositions in terms of both morphology and size; the average particle size is only 727 nm, which is 300 nm smaller than that of pure tungsten powder. The particle sizes in the other compositions do not differ considerably are shown in Figure 3c–e. However, compared with those presented in Figure 3b, these compositions contain many large Al_2_O_3_ particles; this is one of the reasons for the deterioration of the performance with the increase in Al_2_O_3_ content. Figure 3f shows the average particle size of the powders with different compositions. The standard deviations of composite powders with Al_2_O_3_ content from 0 to 0.8 wt.% are 0.2, 0.09, 0.11, 0.12, and 0.12, respectively. The D50 of composite powders with Al_2_O_3_ content from 0 to 0.8 wt.% are 1.05, 0.73, 0.83, 0.94, and 0.93 μm, respectively. The particle size of the powder prepared using the wet chemical method reaches the nanometer level. A distinct difference between the particle size of pure tungsten and the other powders with Al_2_O_3_ can be observed, indicating that adding Al_2_O_3_ effectively reduces the particle size during the hydrothermal synthesis and the reduction process. This has a crucial effect on the subsequent sintering and rolling of the tungsten alloys.

### 3.2. Microstructure of Sintered and Rolled Alloys

**Figure 4:**
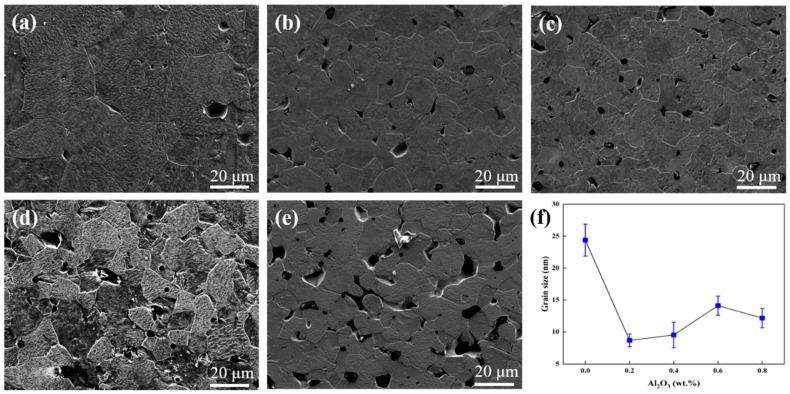
SEM images of the tungsten alloys: (**a**) pure tungsten, (**b**) W-0.2 wt.% Al_2_O_3_, (**c**) W-0.4 wt.% Al_2_O_3_, (**d**) W-0.6 wt.% Al_2_O_3_, (**e**) W-0.8 wt.%Al_2_O_3_, (**f**) grain size of the tungsten alloys.

After the blanks are pressed and sintered and the sample is cut for SEM observation, pores appear in varying degrees in each composition. The reason is that tungsten has a high melting point, and it is difficult to achieve full density even with high temperature and sintering over a long time period. Therefore, the rolling process was adopted to eliminate the pores. The SEM images of the tungsten alloys are shown in Figure 4 and the grain sizes were determined by the linear intercept method. At least 200 intercepts of grains were measured and at least 8 SEM micrographs from random and spread-out locations were analyzed for each condition. As shown in Figure 4a, the grain size of sintered pure tungsten is 24 μm. Figure 4b shows the grain size of sintered W-0.2 wt.%Al_2_O_3_ alloy is only 9 μm. When the content of Al_2_O_3_ is 0.4 wt.%, 0.6 wt.%, and 0.8 wt.% (Figure 4c–e), the grain sizes are 10 μm, 14 μm, and 12 μm, respectively, corresponding to the grain sizes of the alloy powder measured previously. Al_2_O_3_ distributed at the grain boundaries during the sintering process can hinder the diffusion of grain boundaries, thus inhibiting grain growth and achieving grain refinement. Excessive addition of Al_2_O_3_ grains leads to agglomeration, and the ability to inhibit W grain growth is weakened, but the grain size is substantially reduced compared with that of pure tungsten.

**Figure 5:**
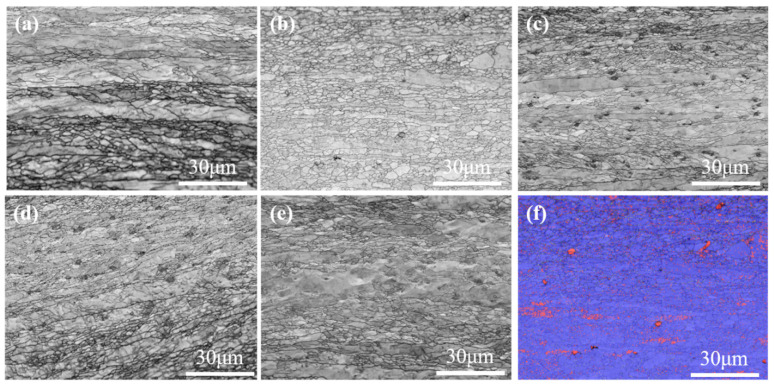
Grain boundaries of the plates: (**a**) pure tungsten, (**b**) W-0.2 wt.% Al_2_O_3_, (**c**) W-0.4 wt.% Al_2_O_3_, (**d**) W-0.6 wt.% Al_2_O_3_, (**e**) W-0.8 wt.% Al_2_O_3_, (**f**) phase distribution of W-0.2 wt.% Al_2_O_3_.

As shown in Figure 5a–e, the grains of the sintered alloy are elongated after rolling. The degrees of elongation and the proportions of large-angle grain boundaries differ for plates with different compositions. Among these, the pure tungsten plate has the largest grains, maximum Feret diameter of 10.2 μm, and smallest percentage (only 39.6%) of large-angle grain boundaries. The W-0.2 wt.% Al_2_O_3_ alloy plate has a maximum Ferret diameter of 6.3 μm and a percentage of large-angle grain boundaries of 48.6%. The remaining plates of the other compositions have no significant advantage in these two aspects. Because of the large plate deformation under extreme stress, many dislocations in the form of dislocation walls and networks are widely distributed in the grain. With the movement of dislocations, a series of interactions between various dislocations cause the dislocation entanglement phenomenon. With the development of this entanglement phenomenon, the grains break into sub-grains; this is why the grains become smaller after rolling. The increase in the dislocation density and sub-grain boundaries gradually increases the deformation resistance of tungsten. The addition of Al_2_O_3_ enhances this phenomenon. Pure tungsten without Al_2_O_3_ impedes dislocations, with long strips of grains appearing after rolling. For several other compositions, aggregation, and uneven distribution due to excessive Al_2_O_3_ addition also cause such phenomena. Thus, the maximum Ferret diameter is the smallest for W-0.2 wt.% Al_2_O_3_. Figure 5f shows the phase distribution of the W-0.2 wt.% Al_2_O_3_ alloy plate. Al_2_O_3_ is distributed more uniformly in this plate compared with those in the plates with other compositions, and the Al_2_O_3_ particles are aggregated to the lowest degree. The uniform distribution of Al_2_O_3_ in the matrix can hinder the migration of grain boundaries during the sintering process, thus inhibiting grain growth. The finer the grains, the smaller the maximum Ferret diameter of the deformed grains. This is the reason for the highest percentage of large-angle grain boundaries, which can influence grain boundary strengthening, and one of the reasons for the higher hardness and compressive strength of the plates with this composition. The plates with other compositions have higher Al_2_O_3_ contents; thus, Al_2_O_3_ tends to aggregate and form large particles. This is why the mechanical properties of the W-0.2 wt.% Al_2_O_3_ alloy plates are better than the properties of the plates with other compositions.

**Figure 6:**
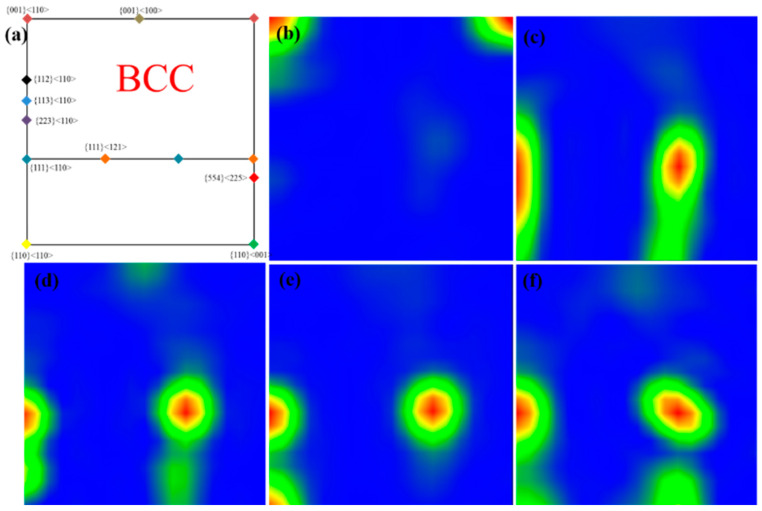
(**a**) standard ODF at φ2 = 45°.ODF diagram for different composition plates, (**b**) pure tungsten, (**c**) W-0.2 wt.% Al_2_O_3_, (**d**) W-0.4 wt.% Al_2_O_3_, (**e**) W-0.6 wt.% Al_2_O_3_, (**f**) W-0.8 wt.% Al_2_O_3_.

**Figure 7:**
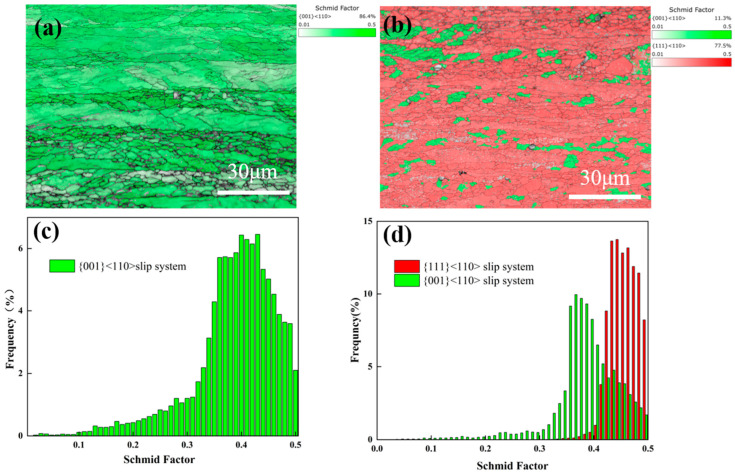
Schmidt coefficient plots and Schmidt coefficient frequency distributions for (**a**,**c**) pure tungsten, (**b**,**d**) W-0.2 wt.% Al_2_O_3_.

As shown in Figure 6a–f, the grain orientation distribution function (ODF) diagram of the alloy plates with different compositions after rolling shows a distinct texture. Tungsten is known to have a body-centered cubic (BCC) structure, the texture of which is usually more obvious at the Euler angle of φ2 = 45°. Figure 6a shows the standard ODF diagram of the BCC structure at the Euler angle of φ2 = 45°. The typical {0 0 1} <1 1 0> BCC plate texture appears in the ODF diagram of pure tungsten plates, and the addition of Al_2_O_3_ transforms the original texture into a {1 1 1} <1 1 0> texture (Figure 6c–f). The strength of the texture structure varies with the change in Al_2_O_3_ content. This knowledge helps us understand the evolution of texture during the rolling process. and allows us to adjust the process parameters and the second phase addition ratio to adapt the texture depending on the application needs. The texture structure mainly depends on the motion of slip and twin systems, and the Schmidt factor diagram theory can explain the evolution of the texture structure. Figure 7 shows the Schmidt factor diagrams for pure tungsten and alloy plates, combined with the orientation distribution function diagrams. The higher Schmidt factor means that the slip system is easier to slip. This is why the {1 1 1} <1 1 0> slip system is easier to open, changing the structure of the plate after adding Al_2_O_3_.

### 3.3. Microstructure and Interface Feature

**Figure 8:**
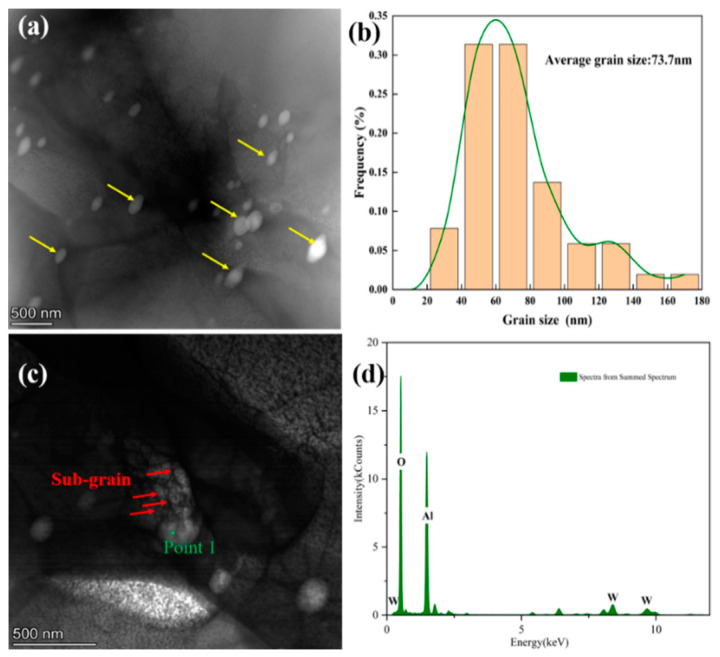
TEM images of the rolled W-0.2 wt.% Al_2_O_3_ plates: (**a**) Al_2_O_3_ present in the tungsten matrix, (**b**) Al_2_O_3_ average grain size, (**c**) sub-boundary around point 1, (**d**) EDS of point 1.

The samples of the rolled plates were subjected to TEM imaging and most of the Al_2_O_3_ grains present in the plates and their fineness are visible in Figure 8a. Figure 8b shows the grain size distribution of the Al_2_O_3_ particles with an average grain size of approximately 73.7 nm. Point 1 in Figure 8c represents many sub-grain boundaries around Al_2_O_3_ particles, as indicated in energy-dispersive X-ray spectroscopy (Figure 8d). This is because Al_2_O_3_, as a high-hardness ceramic material, accumulates around Al_2_O_3_ during the rolling process. The dislocations cannot pass through Al_2_O_3_ during slip. Hence, many dislocations accumulate to form sub-grain boundaries and these sub-grain boundaries strongly impede the movement of dislocations. This is an important reason why Al_2_O_3_ can enhance the strength of the plate.

**Figure 9:**
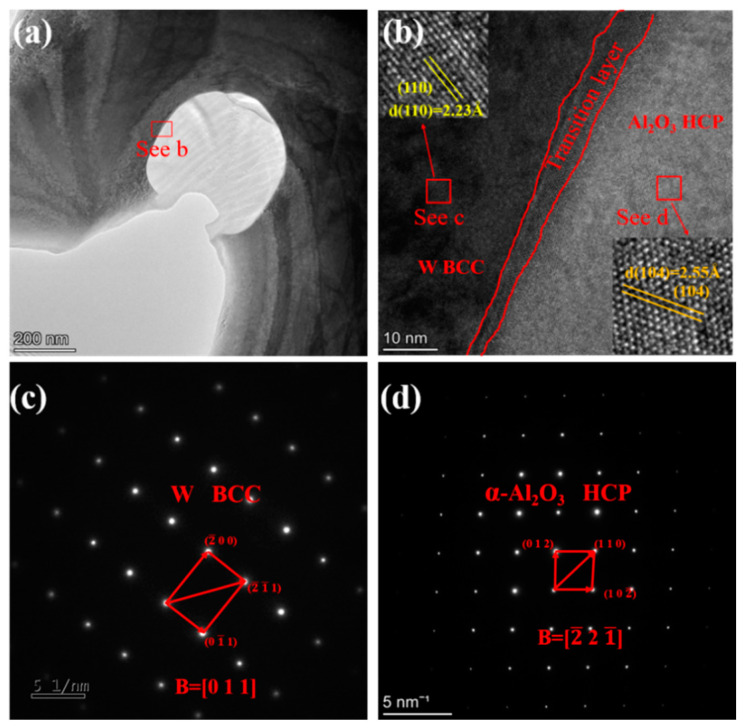
TEM images and selected area electron diffraction patterns analysis of W-0.2 wt.% Al_2_O_3_ plate: (**a**) bright field image, (**b**) HRTEM image of the interface, (**c**) SEAD of tungsten, (**d**) SEAD of Al_2_O_3_.

The selected area electron diffraction (SAED) pattern (Figure 9c) of the tungsten matrix part was analyzed. The crystal plane spacing was 0.159 nm, 0.222 nm, and 0.129 nm, corresponding to the crystal planes of ($\bar{2}$ 0 0), (0 $\bar{1}$ 1), and ($\bar{2}$ $\bar{1}$ 1), respectively. The angular relationship of each crystal plane was verified and determined to be consistent with the (0 1 1) crystal band axis parameter of W. The SAED pattern (Figure 9d) of the Al_2_O_3_ part was analyzed. The crystalline band axis was calculated based on the crystalline plane spacing and angle, and it was consistent with the ($\bar{2}$ 2 $\bar{1}$) crystalline band axis corresponding to α-Al_2_O_3_. Figure 9a shows a clear interface between Al_2_O_3_ and the tungsten matrix. The crystalline plane and the lattice stripe spacing are determined using the Fourier transform, as shown in Figure 9b. The (1 1 0) crystalline plane spacing in the tungsten matrix is 2.23 Å, and the (1 0 4) crystalline plane spacing in α-Al_2_O_3_ is 2.55 Å. According to the crystalline plane mismatch degree formula, the lattice mismatch degree of the two-phase interface is 33%, and the interface is a non-co-grained relationship. As Al_2_O_3_ is a high-hardness inorganic non-metallic material with plasticity very different from that of tungsten, a metallic material, the different deformation ability of the two phases leads to the non-coordinated deformation in the rolling process. A transition layer of approximately 3-5 nm appears at the interface of the two phases after rolling, but no similar transition layer is observed in the sintered state. The Al_2_O_3_ particles are strongly bonded to the W matrix and do not separate during the deformation process (compressive stress state). Therefore, the tungsten grains on the surface layer of the Al_2_O_3_ particles undergo a large slip along the surface of the Al_2_O_3_ particles, causing the tungsten atoms to deviate from their equilibrium position and show amorphous properties. This amorphous transition layer can effectively prevent dislocation movement because amorphous grains do not have many slip systems as in crystals or dense row surfaces in which dislocations can barely move, thus strengthening the tungsten matrix.

### 3.4. Mechanical Properties

**Figure 10:**
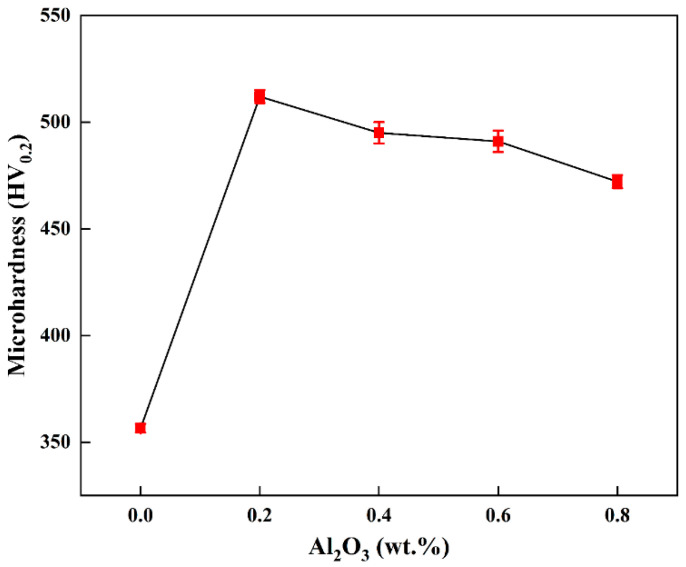
Microhardness of rolled samples with different Al_2_O_3_ contents.

The micro-hardness of tungsten alloy plates with different compositions is shown in Figure 10. The micro-hardness of pure tungsten plate is 356 HV_0.2_, and the micro-hardness of the W-0.2 wt.% Al_2_O_3_ alloy plate is 512 HV_0.2_, which is an increase of 43.7%. However, the micro-hardness of the W-0.4 wt.% Al_2_O_3_ alloy plate decreases to 495 HV_0.2_, and with an increase in the Al_2_O_3_ content, the micro-hardness decreases further. The micro-hardness of the W-0.6 wt.% Al_2_O_3_ alloy plate is 491 HV_0.2_. Among all the tested alloy plates, the microhardness of the W-0.8 wt.% Al_2_O_3_ alloy plate is the lowest (472 HV_0.2_). The SEM images in Figure 4 of the composite powders show the low content of Al_2_O_3_ in the W-0.2 wt.% Al_2_O_3_ and Al_2_O_3_ dispersed around the tungsten grains. After sintering is performed, Al_2_O_3_ is uniformly distributed in the alloy. When subjected to external forces, dislocations are generated inside the alloy, and the fine Al_2_O_3_ particles dispersed inside the alloy play a role in pinning. Al_2_O_3_ particles dispersed in the alloy contribute to nailing dislocations and impede the movement of dislocations. However, when the content of Al_2_O_3_ increases, the fine Al_2_O_3_ particles agglomerate, thereby ineffectively inhibiting the grain growth during the sintering process and reducing the effect of nailing dislocations. The agglomeration of Al_2_O_3_ also results in the inhomogeneous microstructure of the tungsten alloys (see Figure 5), which eventually leads to the decrease in micro-hardness. Therefore, when the Al_2_O_3_ content exceeds a certain limit, the micro-hardness does not continue to increase but rather decreases to values even lower than that of pure tungsten.

**Figure 11:**
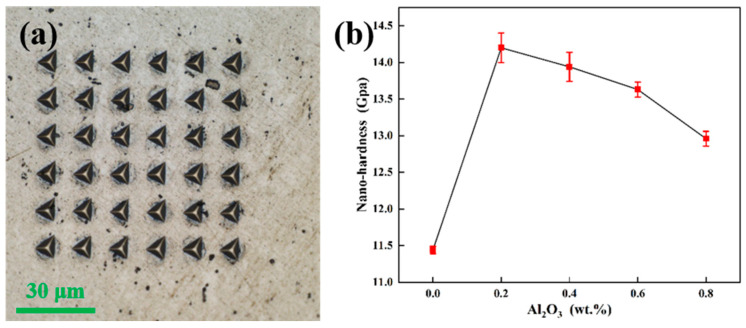
(**a**) Nanoindentation of W-0.2 wt.% Al_2_O_3_ alloy, (**b**) nano-hardness of rolled samples with different Al_2_O_3_ contents.

The nano-indentation characterization technique allows the hardness of microscopic regions within individual grains to be measured and compared with micro-hardness to determine the variation of nano-hardness with indentation depth continuously. Hence, nano-hardness can eliminate the influence of grain boundaries on hardness and thus can directly reflect the effect of microstructure on hardness. Based on previously published work, the minimum indent spacing can be 10 times the indentation depth for a Berkovich tip [24]. In this work, the ratio of indent spacing to indent depth is 10, which can ensure that the data from the nano-indentation tests is valid. Figure 11a shows that the distance between each indentation is moderate and does not affect the test results. Figure 11b shows the nano-hardness of the plates with different compositions. The nano-hardness of pure tungsten is 11.44 GPa. After 0.2 wt.% Al_2_O_3_ is added, and the nano-hardness of the plates reaches 14.2 GPa, which is 24.1% higher than the nano-hardness of pure tungsten. After more Al_2_O_3_ is added, the nano-hardness gradually decreases, but even the lowest is 13.2% higher than that of pure tungsten. The increase in nano-hardness may be due to the interaction mechanism between the dispersed Al_2_O_3_ particles and dislocation motion, mainly the Orowan mechanism. During the downward pressure of the indenter, the dislocations caused by the load are blocked by the dispersed Al_2_O_3_ particles at the grain boundaries, causing an increase in nano-hardness [23]. However, when the Al_2_O_3_ content increases, the fine Al_2_O_3_ particles agglomerate to form large particles, and the uneven distribution reduces the hindering effect of Al_2_O_3_, resulting in a decrease in hardness.

**Figure 12:**
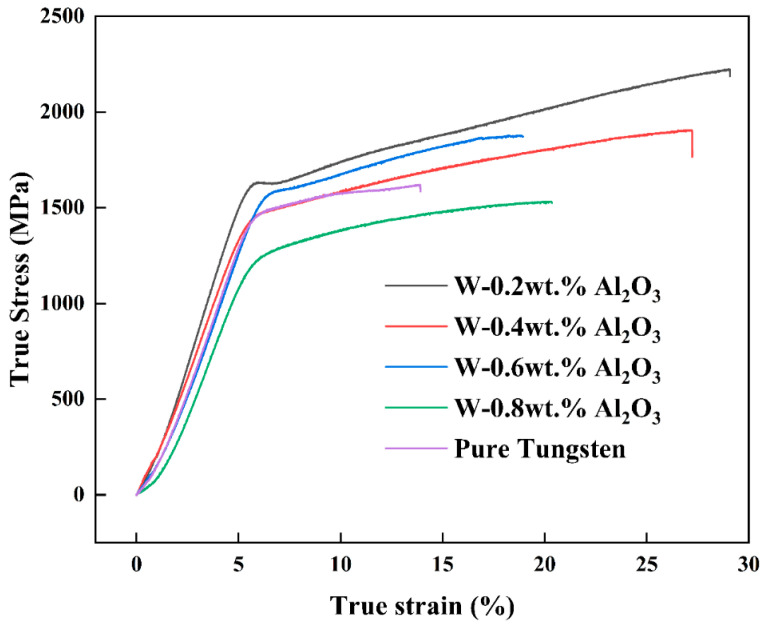
Compressive stress–strain curves of rolled samples with different Al_2_O_3_ contents.

**Figure 13:**
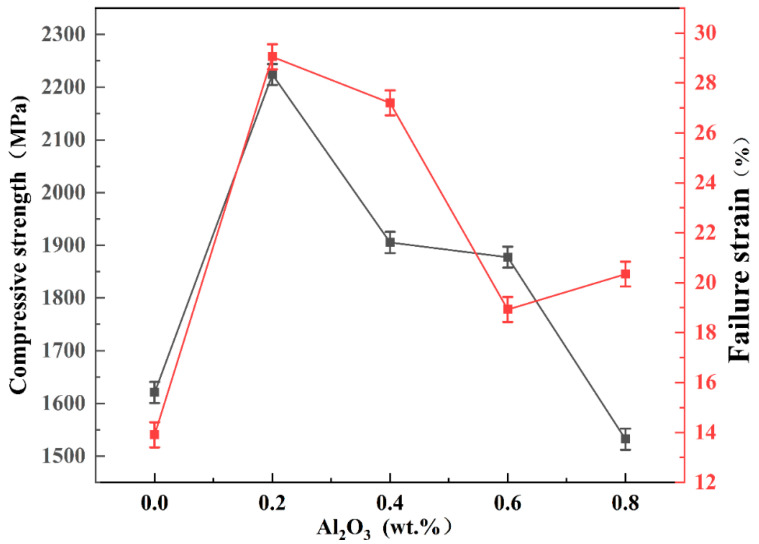
Compressive strength and failure strain of the samples with different Al_2_O_3_ contents.

Figure 12 presents the room-temperature compressive stress–strain curves of tungsten alloy plates with different compositions and the corresponding maximum compressive strength and failure strain are shown in Figure 13. The curves reveal that the compressive strength of pure tungsten is 1620 MPa. The highest compressive strength of 2224 MPa is achieved when 0.2 wt.% Al_2_O_3_ is added, an increase of approximately 603 MPa or 37.2% compared with that of pure tungsten. When the Al_2_O_3_ content reaches 0.8 wt.%, the compressive strength is lower than that of pure tungsten, but the deformation of the W-0.8 wt.% Al_2_O_3_ alloy plates is greater than that of pure tungsten. Pure tungsten fractures at 13.9% deformation, but the fracture of the W-0.8 wt.% Al_2_O_3_ alloy plate occurs when the deformation reaches 20%. The deformation of the W-0.2 wt.% Al_2_O_3_ alloy plates is 29%. In general, the compressive strength is influenced by the Al_2_O_3_ particles. Under stress, the Al_2_O_3_ particles in the tungsten matrix can hinder the movement of dislocations and the expansion of cracks, and at the same time, because the grains are inhibited from growing during sintering and broken into many fine sub-crystals during rolling, the resistance to crack sprouting increases, causing the superior toughness and higher compressive strength of tungsten alloy plates. The compressive strength is about 604 MPa higher than that of pure tungsten plates, and the maximum strain at fracture increases from 13.9% to 29%, improving tungsten’s ductility.

**Figure 14:**
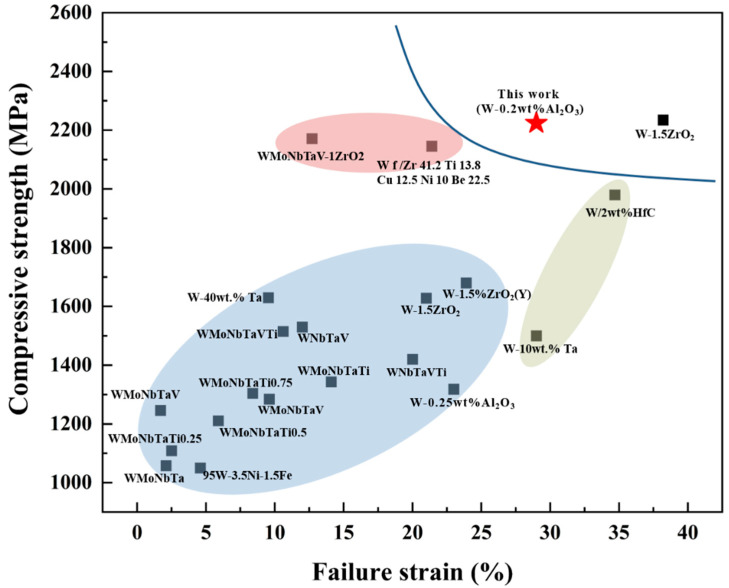
Compressive strength and failure strain diagrams of some tungsten alloys at room temperature.

The mechanical properties of some typical tungsten alloys from the literature are summarized in Table 1 and a visual comparison of these data graphically is shown in Figure 14, indicating that W-2 wt% HfC and W-10 wt.% Ta in the green shaded area has the same or even higher failure strains at room temperature than W-0.2 wt.% Al_2_O_3_ plates, but the strength is more than 240 MPa lower than W-0.2 wt.% Al_2_O_3_ plates. The compressive strength of WMoNbTaV-1ZrO_2_ and W_f_/Zr_41.2_Ti_13.8_Cu_12.5_Ni_10_Be_22.5_ is slightly lower than that of the W-0.2 wt.% Al_2_O_3_ plate, but the failure strain is much lower than that of the W-0.2 wt.% Al_2_O_3_ plates. The rest of the tungsten alloys shaded in blue are far inferior to the W-0.2 wt.% Al_2_O_3_ plates in terms of failure strain and compressive strength. However, compared to ZrO_2_, Al_2_O_3_ is cheaper and can be used to save costs in large batches for industrial production. Comparing the difference in compressive strength and failure strain between spin-forged and sintered W-1.5 ZrO_2_, as well as the significant difference in compressive strength and failure strain between W-0.2 wt.% Al_2_O_3_ sheet and sintered W-0.25 wt.% Al_2_O_3_ alloy of similar composition, it can be seen that forging and extrusion are effective methods to increase the strength and ductility of the material.

## 4. Conclusions

1. Among the alloy powders of different compositions, the average grain size of the W-0.2 wt.% Al_2_O_3_ powder was only 727 nm, while the grain size of the rest of the powders was about 1 μm. The XRD pattern showed no diffraction peaks of Al_2_O_3_ due to the low Al_2_O_3_ content.
2. In the sintered specimens, the average grain size of pure tungsten was 24 μm, and that of W-0.2 wt.% Al_2_O_3_ was only 9 m. The addition of Al_2_O_3_ was effective in refining the grains.
3. The maximum Ferret diameter is 10 μm for pure tungsten and 6 μm for W-0.2 wt.% Al_2_O_3_ plates, with the same size trend as in the sintered state, due to dislocation cleavage of the grains during the rolling process. The texture of the plates changes from {001} <110> to {111} <110> after the addition of Al_2_O_3_.
4. As can be observed in the TEM image, the average grain size of the Al_2_O_3_ in the plate is approximately 100 nm, with a transition layer at the interface between the two phases and a non-co-grained interface relationship.
5. Comparing the mechanical properties of pure tungsten plates with those of tungsten alloy plates of different compositions, the microhardness has increased by up to 156 HV_0.2_, an increase of 43%, while the nano-hardness increased by 2.75 GPa, an increase of 24.1%. The compressive strength of pure tungsten plates was 1620 MPa, but the addition of Al_2_O_3_ increased the compressive strength by up to 600 MPa, an increase of 37%. Pure tungsten plates fractured at a compression deflection of 13.9%, and at an Al_2_O_3_ content of 0.2 wt.%, after which the plates fractured only at a deflection of 29%. This proves that the doping of Al_2_O_3_ has enhanced the strength and toughness of the alloy plates.

## Figures and Tables

**Table 1:** Compressive mechanical properties of typical tungsten alloys at room temperature.

Alloy	State	Compressive Strength	Elongation	References
**95W-3.5Ni-1.5Fe**	Swaged	1434 MPa	4.6%	[25]
**WMoNbTaV**	Sintered	1284.6 MPa	9.6%	[26]
**WMoNbTaV-** **1ZrO_2_**	Sintered	2171.1 MPa	12.7%	[26]
**WMoNbTaV**	Sintered	1246 MPa	1.7%	[27]
**WMoNbTa**	Sintered	1058 MPa	2.1%	[27]
**WMoNbTaTi_0.25_**	Sintered	1109 MPa	2.5%	[28]
**WMoNbTaTi_0.5_**	Sintered	1211 MPa	5.9%	[28]
**WMoNbTaTi_0.75_**	Sintered	1304 MPa	8.4%	[28]
**WMoNbTaTi**	Sintered	1343 MPa	14.1%	[29]
**WMoNbTaVTi**	Sintered	1515 MPa	10.6%	[29]
**WNbTaV**	Sintered	1530 MPa	12%	[30]
**WNbTaVTi**	Sintered	1420 MPa	20%	[30]
**W-1.5 ZrO_2_**	Swaged	2235 MPa	38.2%	[31]
**W-1.5 ZrO_2_**	Sintered	1628 MPa	21%	[32]
**W-1.5% ZrO_2_(Y)**	Sintered	1680 MPa	23.9	[33]
**W_f_/Zr_41.2_Ti_13.8_Cu_12.5_Ni_10_Be_22.5_**	Sintered	2146 MPa	21.4	[34]
**W-40 wt.% Ta**	Sintered	1630 MPa	9.54%	[35]
**W/2 wt.% HfC**	Sintered	1980 MPa	34.7%	[36]
**W-10 wt.% Ta**	Sintered	1500 MPa	29%	[37]
**W-0.25 wt.% Al_2_O_3_**	Sintered	1318 MPa	23%	[15]
**W-0.2 wt.% Al_2_O_3_**	Rolled	2224 MPa	29%	This work
